# Molecular diagnostic challenges for non‐retinal developmental eye disorders in the United Kingdom

**DOI:** 10.1002/ajmg.c.31837

**Published:** 2020-08-23

**Authors:** Daniel Jackson, Samantha Malka, Philippa Harding, Juliana Palma, Hannah Dunbar, Mariya Moosajee

**Affiliations:** ^1^ Moorfields Eye Hospital NHS Foundation Trust London UK; ^2^ UCL Institute of Ophthalmology London UK; ^3^ Great Ormond Street Hospital for Children NHS Trust London UK; ^4^ The Francis Crick Institute London UK

**Keywords:** developmental eye disorders, genetic eye disease, next generation sequencing, targeted gene panels, whole genome sequencing

## Abstract

Overall, approximately one‐quarter of patients with genetic eye diseases will receive a molecular diagnosis. Patients with developmental eye disorders face a number of diagnostic challenges including phenotypic heterogeneity with significant asymmetry, coexisting ocular and systemic disease, limited understanding of human eye development and the associated genetic repertoire, and lack of access to next generation sequencing as regarded not to impact on patient outcomes/management with cost implications. Herein, we report our real world experience from a pediatric ocular genetics service over a 12 month period with 72 consecutive patients from 62 families, and that from a cohort of 322 patients undergoing whole genome sequencing (WGS) through the Genomics England 100,000 Genomes Project; encompassing microphthalmia, anophthalmia, ocular coloboma (MAC), anterior segment dysgenesis anomalies (ASDA), primary congenital glaucoma, congenital cataract, infantile nystagmus, and albinism. Overall molecular diagnostic rates reached 24.9% for those recruited to the 100,000 Genomes Project (73/293 families were solved), but up to 33.9% in the clinic setting (20/59 families). WGS was able to improve genetic diagnosis for MAC patients (15.7%), but not for ASDA (15.0%) and congenital cataracts (44.7%). Increased sample sizes and accurate human phenotype ontology (HPO) terms are required to improve diagnostic accuracy. The significant mixed complex ocular phenotypes distort these rates and lead to missed variants if the correct gene panel is not applied. Increased molecular diagnoses will help to explain the genotype–phenotype relationships of these developmental eye disorders. In turn, this will lead to improved integrated care pathways, understanding of disease, and future therapeutic development.

## 1 INTRODUCTION

Childhood visual impairment has a significant emotional, social and economic impact on the individual, their family, and society as a whole. An estimated 1.4 million children are blind worldwide (Gilbert, 2001), and in the United Kingdom, 1 in 2500 children under the age of 1 year are diagnosed as severely sight impaired with an estimated one‐third having a genetic basis (Rahi and Cable, 2003). The spectrum of developmental eye disorders is vast, and includes microphthalmia, anophthalmia and ocular coloboma (collectively grouped as MAC), anterior segment dysgenesis anomalies (ASDA), congenital cataracts, primary congenital glaucoma (PCG), Leber congenital amaurosis (LCA) and vitreoretinal dysplasia, optic nerve disorders, infantile nystagmus, and albinism (ocular and syndromic). Environmental factors, such as maternal alcohol intake or in utero infections, may cause some of these conditions (Busby, Dolk, & Armstrong, 2005; Chassaing et al., 2014; Givens, Lee, Jones, & Ilstrup, 1993), therefore a detailed prenatal history should be obtained. If an unremarkable pregnancy is reported a genetic basis should be considered.

MAC contributes up to 15% of childhood blindness and severe visual impairment worldwide (Hornby et al., 2000), with a cumulative incidence of 11.9 per 100,000 children (<16 years of age) in the United Kingdom (Shah et al., 2011). A prospective incidence study found that 2% of cases were due to environmental causes, and despite the assumption that the rest were genetic, only 6% of patients received a molecular diagnosis (Shah et al., 2011). MAC patients display significant phenotypic heterogeneity, forming part of a clinical spectrum and mixed phenotypes can often be seen in individuals, for example right microphthalmia with chorioretinal coloboma and left anophthalmia. Other ocular abnormalities, such as ASDA and cataract, can also be found in MAC patients causing a more complex presentation, with 60% having systemic associations (Richardson, Sowden, Gerth‐Kahlert, Moore, & Moosajee, 2017). Over 90 genes linked to MAC have been identified with all forms of inheritance (de novo sporadic, autosomal dominant, autosomal recessive, X‐linked dominant, and X‐linked recessive), demonstrating genetic heterogeneity (Harding & Moosajee, 2019). In severe bilateral cases of microphthalmia and anophthalmia, a genetic cause can be found in up to 80% (Plaisancie, Calvas, & Chassaing, 2016), with heterozygous loss of function variants involving *SOX2* and *OTX2*, and recessive biallelic changes in *STRA6* being the most common (Williamson & FitzPatrick, 2014). However, this represents a small subset of patients with a prevalence of 2–3 per 100,000. For the majority, where asymmetry exists, particularly isolated unilateral cases, the diagnostic rates fall below 10%. Germline mosaicism, nonpenetrance and variable expressivity may be contributing factors (Chassaing et al., 2007; Faivre et al., 2006; Morrison et al., 2002).

The involvement of multiple ocular structures in patients with developmental eye disorders is due to the genes (a significant number being transcription factors) involved in early eye development having a spatiotemporal role in various ocular tissues, thus having a pleiotropic effect if defective. Current genetic testing practice in the United Kingdom for genetically heterogeneous eye conditions utilizes targeted gene panels (e.g., the Oculome; http://www.labs.gosh.nhs.uk/media/764794/oculome_v8.pdf) which encompass known disease‐causing genes that cause both nonsyndromic and syndromic forms of disease. There are exceptions, for example, for children born with aniridia, an array‐CGH is commonly used to detect a deletion involving the *WT1* and *PAX6* genes, if negative then Wilms tumor, aniridia, genitourinary anomalies, and mental retardation (WAGR) syndrome, can be ruled out and single gene PCR‐based sequencing of *PAX6* is undertaken to identify pathogenic variants causing isolated aniridia. It is important to consider that although targeted gene panels, such as the Oculome “Anterior Segment Dysgenesis” panel encompasses related conditions such as ASDA, corneal dystrophies and glaucoma related genes, if a patient also had cataract or coloboma, then the relevant panel may not be selected and the molecular cause missed. Hence accurate phenotypic descriptions using Human Phenotype Ontology (HPO) terms must be given so the clinical scientists can consider the differential genes that may be involved and apply multiple gene panels if necessary. Whole genome sequencing (WGS) remains a research‐based test in the United Kingdom but is transitioning to clinical accreditation, however, similar principles will apply to selecting the correct panel of genes to be screened based on phenotype.

In contrast, inherited retinal disorders (IRDs), although considered phenotypically heterogenous, commonly have a symmetrical appearance with an onset after birth through to late adulthood which can be monitored closely with state‐of‐the‐art retinal imaging. Over 250 disease‐causing genes have been identified, mainly over the past two decades, and this had led to the first approved retinal gene therapy, voretigene neparovec, for autosomal recessive biallelic *RPE65*‐retinopathy, with a multitude of gene/mutation‐based clinical trials underway (Maguire et al., 2019; Miraldi Utz, Coussa, Antaki, & Traboulsi, 2018; Russell et al., 2017). Genetic diagnostic rates in IRDs vary according to the population being tested, but range between 50 and 70% (Audo et al., 2012; Bernardis et al., 2016; Consugar et al., 2015; Ellingford et al., 2016; Jiman et al., 2020; Tayebi et al., 2019). The progress seen in the IRD field is likely due to the consistent scientific investment made internationally. A PubMed search on 16th May 2020 of papers relating to IRDs with search terms “retinal dystrophy” came to 12,953, whereas those for “developmental eye disorders” was 7,689; “anterior segment dysgenesis” 2,402; “microphthalmia” 4,837; “anophthalmia” 1,612; coloboma 5,152; and “congenital cataract” 5,793.

In this study, we report our real‐world clinical experience of genetic testing of pediatric patients with developmental eye disorders, excluding IRDs, vitreoretinopathies and optic nerve disorders (including hereditary optic neuropathies). We compare our rates of diagnosis to published studies, using current clinically accredited targeted gene panels and research‐based whole genome sequencing (WGS) tests, for patients that presented to the ocular genetics service at Moorfields Eye Hospital NHS Foundation Trust (MEH), which oversees the care of the largest number of genetic eye disease patients of any one site in the United Kingdom.

## 2 METHODS

### 2.1 Editorial policies and ethical considerations

This study had relevant local and national research ethics committee approvals (MEH and the Northwest London Research Ethics Committee), and adhered to the tenets of the Declaration of Helsinki. Patients and relatives gave written informed consent for genetic testing through either the Genetic Study of Inherited Eye Disease (REC reference 12/LO/0141) or Genomics England 100,000 Genomes project (REC reference 14/EE/1112).

### 2.2 Genetic screening methods

Only families with nonsyndromic and syndromic microphthalmia, anophthalmia, ocular coloboma (MAC), anterior segment dysgenesis anomalies (ASDA) including primary congenital glaucoma, corneal dystrophies, and aniridia, congenital cataract, infantile nystagmus and albinism were included in this analysis. Consecutive patients presenting to the pediatric ocular genetics service at MEH between 1st October 2017 and 30th September 2018 were investigated. In addition, the cohort of patients with corresponding diagnoses recruited into the UK Genomics England 100,000 Genomes Project (Turnbull et al., 2018) from 2015 to 2018 at MEH were also scrutinized.

Molecular testing was performed in the clinical and research setting, using targeted gene panel testing (Oculome; http://www.labs.gosh.nhs.uk/media/764794/oculome_v8.pdf) through the Rare & Inherited Disease Genomic Laboratory at Great Ormond Street Hospital (London, UK) and whole genome sequencing (WGS) as part of the UK Genomics England 100,000 Genomes Project, for which results were reviewed by a multidisciplinary team (including molecular biologists, clinical geneticists, as well as the ophthalmology specialist managing the family), to confirm variant pathogenicity, prevalence in publicly available genome databases, the clinical phenotype and mode of inheritance, before the molecular diagnosis was established. The datasets (variants) generated for this study were submitted to ClinVar (https://www.ncbi.nlm.nih.gov/clinvar/) through the Rare & Inherited Disease Genomic Laboratory at Great Ormond Street Hospital (London, UK).

## 3 RESULTS

### 3.1 Real world genetic outcomes for patients with developmental eye disorders

**FIGURE 1:**
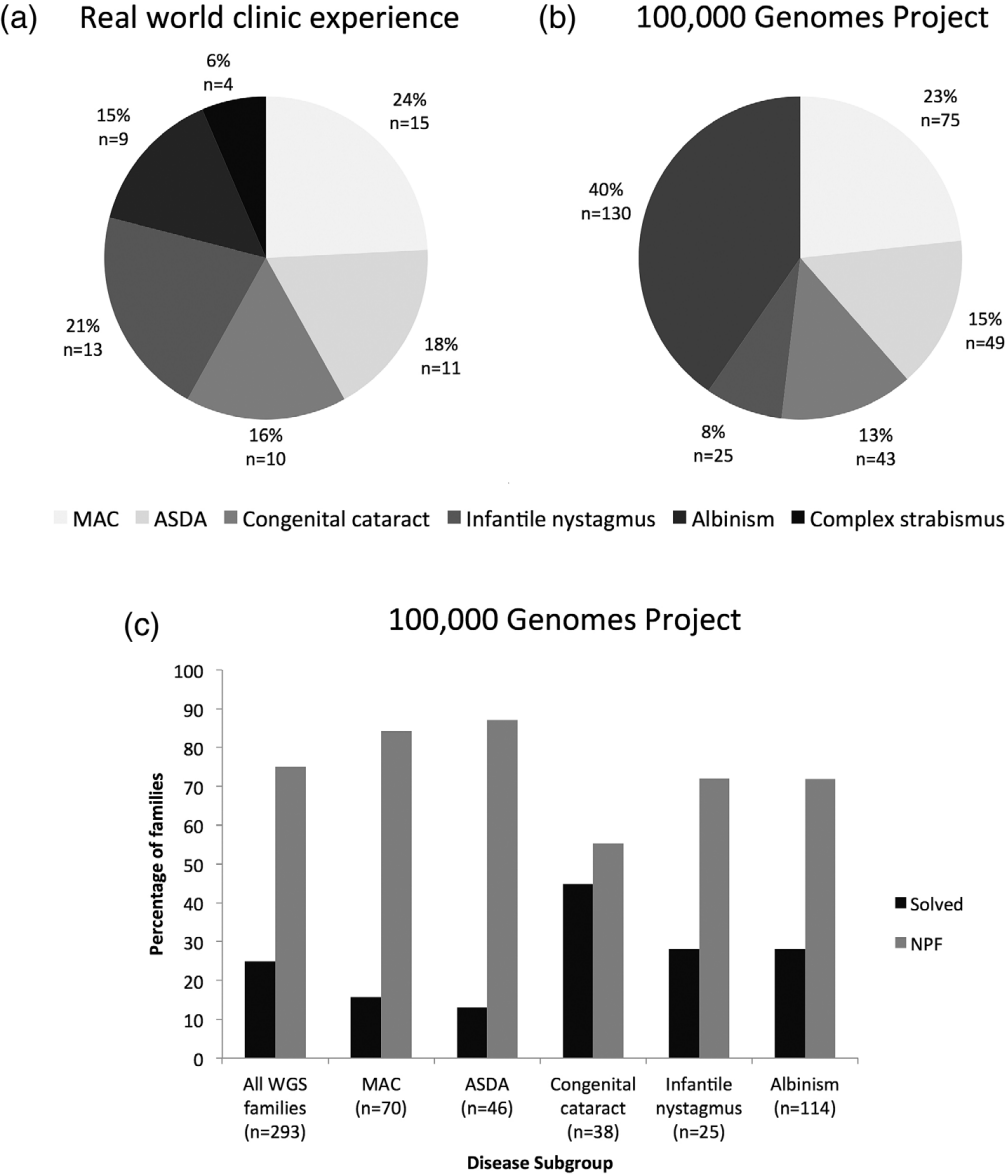
Overview of disease subgroups presenting to a pediatric ocular genetics service and those recruited into the UK 100,000 Genomes Project with corresponding diagnostic rates. (a) Proportion of disease subgroups of developmental eye disorder families seen through the pediatric ocular genetics service between 1st October 2017 and 30th September 2018 at MEH (Moorfields Eye Hospital NHS Foundation Trust). (b) Proportion of disease subgroups of families with developmental eye disorders recruited into the 100,000 Genomes Project between 2015 and 2018. (c) Molecular diagnostic rates from whole genome sequencing (WGS) by disease subgroup for families recruited into the 100,000 Genomes Project. ASDA, anterior segment dysgenesis anomalies; MAC, microphthalmia, anophthalmia and coloboma; NPF, no primary findings

A retrospective observational study identified 72 consecutive patients from 62 families with developmental eye disorders, who attended the pediatric ocular genetics service at MEH over a 12‐month period. The families were divided into 15 MAC (24.2%), 11 ASDA (17.7%, including one aniridia, three corneal dystrophies and two glaucoma [congenital and juvenile‐onset]), 10 congenital cataracts (16.1%), 13 infantile nystagmus (21.0%), nine albinism (14.5%) and four complex strabismus patients (6.5%, including two congenital fibrosis of extraocular muscles, one Duane syndrome, and one with query blepharophimosis, ptosis and epicanthus inversus syndrome) (Figure 1a). The mean ± *SD* age of children was 4.9 ± 4.2 years (range 1 month‐15 years) with 41.7% (*n* = 30) being female. The ethnicity of patients was divided into 18 White British (25%), 11 White other (15.3%), five Asian Pakistani (6.9%), three Asian Bangladeshi (4.2%), three Asian Indian (4.2%), six Asian other (8.3%), two Black African (2.8%), one Black Caribbean (1.4%), two Black other (2.8%), two mixed White and Black African (2.8%), 11 other ethnicities (15.3%), and eight were not stated (11.1%). All patient demographics including clinical and genetic details are listed in Supplementary Table S1.

In total, 69 patients from 59 families (95.2%) proceeded with genetic testing following informed consent, two patients declined, and it was not possible to obtain a sample from one patient. Of the 59 families who consented, 13 had a targeted gene panel (22.0%), 45 had WGS through the 100,000 Genomes Project (76.3%) and one family had single gene test for *PAX6*. Most families opted for WGS due to the superiority of the test and its coverage despite it being on a research basis. Those that underwent a targeted gene panel did so as they either did not meet the 100,000 Genomes Project study eligibility criteria, did not want to partake in a research study, or were concerned about the length of time to get results for family planning purposes.

Overall, 29 patients from 20 families (42.0% of tested patients; 33.9% of tested families) received a molecular diagnosis (Table 1). For the disease subgroups, the following molecular diagnostic rates were achieved (based on number of families undergoing genetic testing); 21.4% MAC (3/14), 60% ASDA (6/10), 44.4% congenital cataracts (4/9), 15.4% infantile nystagmus (2/13), 55.6% albinism (5/9) and nil for the complex strabismus disorders (0/4). If the ASDA group is subdivided; 60% ASDA (3/5), 66.7% corneal dystrophies (2/3) and 50% glaucoma (1/2). Of the solved families, 5/20 had targeted gene panel testing (25%) and 14/20 had WGS (70%), and one had single gene testing (5%). Of the 36 families that received a no primary finding result, seven were from panel test and 29 from WGS, suggesting that overall, those that had a panel test had a 46.2% (6/13) diagnostic rate and for those with WGS was 35.6% (16/45), although this is not a valid comparison due to differing indications and sample size.

**TABLE 1:** Variant details and confirmed phenotype for solved families attending the ocular genetics service

Family ID	Disease group	Test	Gene	Confirmed phenotype (OMIM)	Zygosity	Variant	Variant type	Inheritance
1	MAC	Panel	*KMT2D*	Kabuki syndrome 1	Het	c.6354del; p.Ala2119Leufs*25	Frameshift deletion	AD
4	MAC	WGS	*MAB21L2*	Microphthalmia/coloboma and skeletal dysplasia syndrome	Het	c.379A>T; p.Lys127Ter	Nonsense	AD
9	MAC	WGS	*ALDH1A3*	Microphthalmia, isolated 8	Hom	c.104T>C; p.(Phe35Ser)	Missense	AR
16	ASDA	Panel	*SLC4A11*	Corneal endothelial dystrophy, autosomal recessive	Het	c.2321+1G>A	Noncoding (splice)	AR
					Het	c.508G>A; p.(Glu170Lys)	Missense	
17	ASDA	WGS	*KERA*	Cornea plana 2, autosomal recessive	Hom	c.809C>T; p.(Ser270Leu)	Missense	AR
20	ASDA	Single gene	*PAX6*	Aniridia	Het	c.1253_1262del10; p.(Trp418TyrfsTer104)	Frameshift deletion	AD
21	ASDA	Panel	*KERA*	Cornea plana 2, autosomal recessive	Hom	c.528C>G; p.(Asn176Lys)	Missense	AR
22	ASDA	Panel	*TGFB1*	Reis–Bucklers corneal dystrophy	Het	c.1664G>A; p.(Arg555Gln)	Missense	AD
25	ASDA	Panel	*MYOC*	Glaucoma 1A, primary open angle	Het	c.760C>A; p.(Pro254Thr)	Missense	AD
27	Cataract	WGS	*CRYBB2*	Cataract 3, multiple types	Het	c.463C>T; p.(Gln155*)	Nonsense	AD
28	Cataract	WGS	*EPHA2*	Cataract 6, multiple types	Het	c.1751C>T; p.Pro584Leu	Missense	AD
32	Cataract	WGS	*HSF4*	Cataract 5, multiple types	Het	c.360+1G>A	Noncoding (splice)	AD
36	Cataract	WGS	*BCOR*	Congenital cataract	Het	c.856del; p.(Ser286Alafs*92)	Frameshift deletion	XD
40	Nystagmus	WGS	*CACNA1A*	Idiopathic infantile nystagmus	Het	c.2566G>T; p.(Glu856*)	Nonsense	AD
46	Nystagmus	WGS	*FRMD7*	Nystagmus 1, congenital, X‐linked	Hemi	c.383‐1G>A	Noncoding (splice)	XR
51	Albinism	WGS	*OCA2*	Albinism, oculocutaneous, type II	Het	c.619_636del; p.(Leu207_Leu212del)	Inframe deletion	AR
					Het	c.1327G>A; p.(Val443Ile)	Missense	
52	Albinism	WGS	*GPR143*	Nystagmus 6, congenital, X‐linked	Hemi	c.11C>G; p.(Pro4Arg)	Missense	XR
53	Albinism	WGS	*HPS6*	Hermansky–Pudlak syndrome 6	Hom	c.1228_1252del; p.(Tyr410Valfs*9)	Frameshift deletion	AR
56	Albinism	WGS	*SLC38A8*	Foveal hypoplasia 2, with or without optic nerve misrouting and/or anterior segment dysgenesis	Het	c.435G>A; p.Trp145Ter	Nonsense	AR
					Het	c.632+1G>A	Noncoding (splice)	
58	Albinism	WGS	*SLC38A8*	Foveal hypoplasia 2, with or without optic nerve misrouting and/or anterior segment dysgenesis	Hom	c.264C>G; p.Tyr88*	Nonsense	AR

Pathogenic variants were found in 19 distinct genes. The genes identified for MAC were *KMT2D*, *MAB2IL2*, *ALDH1A3*; ASDA were *KERA*, *PAX6*, *MYOC*, *TGFBI*, and *SLC4A11*; congenital cataract were *CRYBB2*, *EPHA2*, *HSF4* and *BCOR*; infantile nystagmus were *CACNA1A* and *FRMD7*; and albinism were *OCA2*, *GPR143*, *HPS6* and *SLC38A8* (Table 1).

### 3.2 Outcomes for developmental eye disorders through whole genome sequencing

As the aforementioned consecutive cohort of patients had a mixed range of genetic testing, we looked at all developmental eye disorder patients with the same disease criteria who had been recruited into the 100,000 Genomes Project between 2015 and 2018 and received WGS as a gold standard. A total of 322 families were recruited, divided into 75 MAC (23.3%), 49 ASDA (15.2%, including three aniridia, two corneal dystrophies, and 42 primary congenital glaucoma), 43 congenital cataracts (13.4%), 25 infantile nystagmus (7.8%), 130 albinism (40.4%) (Figure 1b).

Two hundred and ninety‐three families have received their results, with 29 still pending. Of this 73 families (24.9%) received a molecular diagnosis but 220 (75.1%) had no primary findings (Table 2). For the disease subgroups the following molecular diagnostic rates were achieved; 15.7% MAC (11/70), 13.0% ASDA (6/46, but 12.8% for primary congenital glaucoma [5/39]), 44.7% congenital cataracts (17/38), 28.0% infantile nystagmus (7/25), and 28.1% albinism (32/114) (Figure 1c).

**TABLE 2:** Variant details and confirmed phenotype for solved families who underwent WGS

Family ID	Ethnicity	Disease group	Gene	Confirmed phenotype (OMIM)	Zygosity	Variant	Variant type	Inheritance
9	Asian Pakistani	MAC	*ALDH1A3*	Microphthalmia, isolated 8	Hom	c.104T>C; p.(Phe35Ser)	Missense	AR
26105	White British	MAC	*CREBBP*	Rubinstein–Taybi syndrome 1	Het	c.2308_2315dup; p.(Pro773Leufs*6)	Frameshift deletion	AD
26334	White British	MAC	*GJA8*	Cataract 1, multiple types	Het	c.290T>G; p.(Val97Gly)	Missense	AD
17	Asian Pakistani	MAC	*KERA*	Cornea plana 2, autosomal recessive	Hom	c.809C>T; p.(Ser270Leu)	Missense	AR
4	Asian Indian	MAC	*MAB21L2*	Microphthalmia/coloboma and skeletal dysplasia syndrome	Het	c.379A>T; p.Lys127Ter	Nonsense	AD
21143	Asian Pakistani	MAC	*MFRP*	Microphthalmia, isolated 5	Hom	c.1150del; p.(His384Thrfs*94)	Frameshift deletion	AR
25272	White other	MAC	*PAX6*	Coloboma, ocular	Het	c.413A>G; p.(Asn138Ser)	Missense	AD
16899	Black African	MAC	*PRSS56*	Microphthalmia, isolated 6	Hom	c.320G>A; p.(Gly107Glu)	Missense	AR
25356	White British	MAC	*PRSS56*	Microphthalmia, isolated 6	Het	c.1439G>C; p.(Arg480Pro)	Missense	AR
					Het	c.1573del; p.(Val525Cysfs*55)	Frameshift deletion	
13302	Not stated	MAC	*PTPN11*	Noonan syndrome 1	Het	c.417G>C; p.(Glu139Asp)	Missense	AD
23756	White other	MAC	*TFAP2A*	Branchiooculofacial syndrome	Het	c.890C>A; p.(Ala297Asp)	Missense	AD
25755	Mixed: White/Asian	ASDA (aniridia)	*PAX6*	Aniridia	Het	c.112delG; p.Arg38GlyfsTer30	Frameshift deletion	AD
16024	Asian Pakistani	ASDA (glaucoma)	*CYP1B1*	Anterior segment dysgenesis 6, multiple subtypes	Hom	c.1169G>A; p.(Arg390His)	Missense	AR
23974	White British	ASDA (glaucoma)	*CYP1B1*	Anterior segment dysgenesis 6, multiple subtypes	Het	c.171G>A; p.(Trp57*)	Nonsense	AR
					Het	c.1147G>A; p.(Ala383Thr)	Missense	
24231	White British	ASDA (glaucoma)	*CYP1B1*	Anterior segment dysgenesis 6, multiple subtypes	Het	c.840C>A; p.(Cys280*)	Nonsense	AR
					Het	c.1168C>T; p.(Arg390Cys)	Missense	
26098	White British	ASDA (glaucoma)	*CYP1B1*	Anterior segment dysgenesis 6, multiple subtypes	Hom	c.1345del; p.(Asp449Metfs*8)	Frameshift deletion	AR
25760	White British	ASDA (glaucoma)	*FOXC1*	Anterior segment dysgenesis 3, multiple subtypes	Het	c.1009_1012dup; p.(Ala338GlyfsTer191)	Frameshift duplication	AD
27	Asian other	Cataract	*CRYBB2*	Cataract 3, multiple types	Het	c.463C>T; p.(Gln155*)	Nonsense	AD
26275	White British	Cataract	*CRYBB2*	Cataract 3, multiple types	Het	c.547C>T; p.(Gln183*)	Nonsense	AD
23145	Asian Pakistani	Cataract	*CRYBB3*	Cataract 22	Het	c.466G>A; p.(Gly156Arg)	Missense	AD
23076	White British	Cataract	*CRYGD*	Cataract 4, multiple types	Het	c.418C>T; p.(Arg140*)	Nonsense	AD
26301	Black African	Cataract	*CRYGD*	Cataract 4, multiple types	Het	c.70C>A; p.Pro24Thr	Missense	AD
28	White British	Cataract	*EPHA2*	Cataract 6, multiple types	Het	c.1751C>T; p.Pro584Leu	Missense	AD
26333	Other	Cataract	*GJA8*	Cataract 1, multiple types	Het	c.134G>T; p.(Trp45Leu)	Missense	AD
32	Other	Cataract	*HSF4*	Cataract 5, multiple types	Het	c.360+1G>A	Noncoding (splice)	AD
25141	White British	Cataract	*MAF*	Cataract 21, multiple types	Het	c.782T>C; p.(Phe261Ser)	Missense	AD
26351	White British	Cataract	*MIP*	Cataract 15, multiple types	Het	c.519del; p.(Phe174Leufs*10)	Frameshift deletion	AD
12720	Asian Bangladeshi	Cataract	*TDRD7*	Cataract 36	Hom	c.1591C>T; p.(Arg531Trp)	Missense	AR
36	White British	Cataract	*BCOR*	Congenital cataract	Het	c.856del; p.(Ser286Alafs*92)	Frameshift deletion	XD
22026	White other	Cataract	*CHMP4B*	Cataract 31, multiple types	Het	c.481G>C; p.Glu161Gln	Missense	AD
23303	Other	Cataract	*CRYBB2*	Cataract 3, multiple types	Het	c.355G>A; p.(Gly119Arg)	Missense	AD
24533	White British	Cataract	*NHS*	Nance–Horan syndrome	Hemi	c.245dup; p.(Pro83Alafs*100)	Frameshift duplication	XR
23139	White British	Cataract	*PITX3*	Cataract 11, multiple types	Het	c.785C>A; p.(Ser262*)	Nonsense	AD
26297	Asian Indian	Cataract	*CRYBB1*	Cataract 17, multiple types	Het	c.667C>T; p.(Gln223*)	Nonsense	AD
40	Mixed White/Black African	Nystagmus	*CACNA1A*	Idiopathic infantile nystagmus	Het	c.2566G>T; p.(Glu856*)	Nonsense	AD
10282	White British	Nystagmus	*FRMD7*	Nystagmus 1, congenital, X‐linked	Hemi	c.206‐5T>A	Noncoding (splice)	XR
18284	White British	Nystagmus	*FRMD7*	Nystagmus 1, congenital, X‐linked	Hemi	c.1003C>T p.(Arg335*)	Nonsense	XR
26131	Mixed: Other	Nystagmus	*FRMD7*	Nystagmus 1, congenital, X‐linked	Hemi	c.875T>C; p.(Leu292Pro)	Missense	XR
46	Other	Nystagmus	*FRMD7*	Nystagmus 1, congenital, X‐linked	Hemi	c.383‐1G>A	Noncoding (splice)	XR
25824	Black Caribbean	Nystagmus	*OCA2*	Albinism, oculocutaneous, type II	Het	c.1103C>T; p.(Ala368Val)	Missense	AR
					Het	c.619_636del; p.(Leu207_Leu212del)	Inframe deletion	
26350	Asian Pakistani	Nystagmus	*SLC38A8*	Foveal hypoplasia 2, with or without optic nerve misrouting and/or anterior segment dysgenesis	Hom	c.264C>G; p.Tyr88*	Nonsense	AR
52	Black Caribbean	Albinism	*GPR143*	Nystagmus 6, congenital, X‐linked	Hemi	c.11C>G; p.(Pro4Arg)	Missense	XR
14259	White British	Albinism	*GPR143*	Ocular albinism, type I	Hemi	c.238‐240del, p.Leu80del	Inframe deletion	XR
17466	Asian Indian	Albinism	*GPR143*	Ocular albinism, type I	Hemi	c.251‐1G>C	Noncoding (splice)	XR
18142	Asian Indian	Albinism	*GPR143*	Ocular albinism, type I	Hemi	c.499del; p.(Leu167Cysfs*51)	Frameshift deletion	XR
18524	White British	Albinism	*GPR143*	Ocular albinism, type I	Hemi	c.874T>G; p.Trp292Gly	Missense	XR
20738	White British	Albinism	*GPR143*	Ocular albinism, type I	Hemi	c.703G>A; p.Glu235Lys	Missense	XR
24336	White British	Albinism	*GPR143*	Ocular albinism, type I	Hemi	c.180dup; p.(Ala61Argfs*40)	Frameshift duplication	XR
25692	Black African	Albinism	*GPR143*	Ocular albinism, type I	Hemi	c.691T>C; p.Tyr231His	Missense	XR
53	Other	Albinism	*HPS6*	Hermansky–Pudlak syndrome 6	Hom	c.1228_1252del; p.(Tyr410Valfs*9)	Frameshift deletion	AR
51	White British	Albinism	*OCA2*	Albinism, oculocutaneous, type II	Het	c.619_636del; p.(Leu207_Leu212del)	Inframe deletion	AR
					Het	c.1327G>A; p.(Val443Ile)	Missense	
14205	White British	Albinism	*OCA2*	Albinism, oculocutaneous, type II	Hom	c.1320G>C; p.(Leu440Phe)	Missense	AR
22452	Black African	Albinism	*OCA2*	Albinism, oculocutaneous, type II	Het	c.1182+1G>A	Noncoding (splice)	AR
					Het	c.2079G>A; p.(Glu693=)	Synonymous	
24075	Black African	Albinism	*OCA2*	Albinism, oculocutaneous, type II	Hom	c.619_636del; p.(Leu207_Leu212del)	Inframe deletion	AR
25242	White British	Albinism	*OCA2*	Albinism, oculocutaneous, type II	Het	c.1320G>C; p.(Leu440Phe)	Missense	AR
					Het	c.1327G>A; p.(Val443Ile)	Missense	
25246	White other	Albinism	*OCA2*	Albinism, oculocutaneous, type II	Het	c.1286T>C; p.(Leu429Pro)	Missense	AR
					Het	c.1327G>A; p.(Val443Ile)	Missense	
26332	White British	Albinism	*OCA2*	Albinism, oculocutaneous, type II	Het	c.1465A>G; p.(Asn489Asp)	Missense	AR
					Het	c.1503+5G>A	Noncoding (splice)	
20483	Asian Pakistani	Albinism	*SLC24A5*	Albinism, oculocutaneous, type VI	Hom	c.568_572del; p.(Ile190*)	Nonsense	AR
56	White British	Albinism	*SLC38A8*	Foveal hypoplasia 2, with or without optic nerve misrouting and/or anterior segment dysgenesis	Het	c.435G>A; p.Trp145Ter	Nonsense	AR
					Het	c.632+1G>A	Noncoding (splice)	
58	Asian Indian	Albinism	*SLC38A8*	Foveal hypoplasia 2, with or without optic nerve misrouting and/or anterior segment dysgenesis	Hom	c.264C>G; p.Tyr88*	Nonsense	AR
26364	Asian other	Albinism	*SLC38A8*	Foveal hypoplasia 2, with or without optic nerve misrouting and/or anterior segment dysgenesis	Hom	c.698A>G; p.(Glu233Gly)	Missense	AR
12189	Asian Indian	Albinism	*TYR*	Albinism, oculocutaneous, type IA	Hom	c.1255G>A; p.(Gly419Arg)	Missense	AR
12676	White British	Albinism	*TYR*	Albinism, oculocutaneous, type IA	Het	c.832C>T; p.(Arg278*)	Nonsense	AR
					Het	c.232G>T; p.(Glu78*)	Nonsense	
18849	White other	Albinism	*TYR*	Albinism, oculocutaneous, type IA	Hom	c.371T>G; p.(Phe124Cys)	Missense	AR
20104	Mixed: White/Black Caribbean	Albinism	*TYR*	Albinism, oculocutaneous, type IA	Het	c.241C>T; p.(Pro81Ser)	Missense	AR
					Het	c.1217C>T; p.(Pro406Leu)	Missense	
22206	White British	Albinism	*TYR*	Albinism, oculocutaneous, type IA	Het	c.823G>T; p.(Val275Phe)	Missense	AR
					Het	c.1118C>A; p.(Thr373Lys)	Missense	
22894	White Irish	Albinism	*TYR*	Albinism, oculocutaneous, type IA	Het	c.823G>T; p.(Val275Phe)	Missense	AR
					Het	c.1118C>A; p.(Thr373Lys)	Missense	
23431	Asian Indian	Albinism	*TYR*	Albinism, oculocutaneous, type IA	Hom	c.832C>T; p.(Arg278*)	Nonsense	AR
23561	White Irish	Albinism	*TYR*	Albinism, oculocutaneous, type IA	Het	c.1336G>A; p.(Gly446Ser)	Missense	AR
					Het	c.1118C>A; p.(Thr373Lys)	Missense	
25284	White other	Albinism	*TYR*	Albinism, oculocutaneous, type IA	Het	c.1357C>T; p.(Gln453*)	Nonsense	AR
					Het	c.1099C>T; p.(His367Tyr)	Missense	
25444	White British	Albinism	*TYR*	Albinism, oculocutaneous, type IA	Het	c.1037‐1G>A	Noncoding (splice)	AR
					Het	c.1118C>A; p.(Thr373Lys)	Missense	
25451	White British	Albinism	*TYR*	Albinism, oculocutaneous, type IA	Het	c.1118C>A; p.(Thr373Lys)	Missense	AR
					Het	c.1205G>A; p.(Arg402Gln)	Missense	
					Hom	c.575C>A; p.(Ser192Tyr)	Missense	
25959	White British	Albinism	*TYR*	Albinism, oculocutaneous, type IA	Het	c.229C>T; p.(Arg77Trp)	Missense	AR
					Het	c.1205G>A; p.(Arg402Gln)	Missense	

Pathogenic variants were found in 38 distinct genes. Those identified for MAC were *PRSS56* (two families), *ALDH1A3*, *CREBBP*, *GJA8*, *KERA*, *MAB21L2*, *MFRP*, *PAX6*, *PTPN11*, and *TFAP2A*; ASDA were *CYP1B1* (four families), *FOXC1* and *PAX6*; congenital cataract were *CRYBB2* (three families), *CRYGD* (two families), *BCOR*, *CRYBB1*, *CRYBB3*, *CHMP4B*, *EPHA2*, *GJA8*, *HSF4*, *MAF*, *MIP*, *NHS*, *PITX3*, and *TDRD7*; infantile nystagmus were *FRMD7* (four families), *OCA2*, *SLC38A8*, *CACNA1A*; and albinism were *TYR* (12 families), *GPR143* (eight families), *OCA2* (seven families), *SLC38A8* (three families), *HPS6*, and *SLC24A5* (Table 2). WGS seeks to screen both coding and noncoding regions of the gene, but only 8 out of 93 (8.6%) variants were found to be noncoding (all were splice‐site mutations); these were found in one cataract patient (*HSF4*), two nystagmus patients (both *FRMD7*), and five albinism patients (*OCA2* [two patients], *GPR143*, *SLC38A8*, and *TYR*). Noncoding variants were only found in splice regions due to the limitations of the UK Genomics England 100,000 Genomes Project diagnostic pipeline. As with clinically accredited diagnostic targeted gene panels (e.g., the Oculome), the focus was on the detection of class 4 and 5 variants. So while WGS has the capacity to detect all noncoding variation, only those with a canonical splicing effect or those previously identified and/or functionally proven variants will be regarded as class 4 or 5. For unsolved cases, the discovery of novel noncoding variants is undertaken by further data mining in the research setting.

## 4 DISCUSSION

The spectrum of genetic and phenotypic heterogeneity, and copresentation of developmental eye disorders is a major challenge in obtaining a molecular diagnosis. A mixed testing approach in the clinic using targeted gene panels, single gene and WGS yielded a higher overall molecular diagnostic rate of 33.9% (20 out of 59 families) compared to WGS alone. Arguably, only 24.9% received a result from the 100,000 Genomes Project (73 out of 293 families), but this involved a larger sample size and was in‐line with other studies. The development of a next generation sequencing (NGS) panel assay for ocular conditions known as the Oculome panel test (http://www.labs.gosh.nhs.uk/media/764794/oculome_v8.pdf) screened 277 pediatric patients across several panels (including MAC, ASDA, cataract, retinal and albinism) with 68 individuals (24.5%) receiving a definitive diagnosis (Patel et al., 2019). For disease subgroups, there are significant differences between the published outcomes and what we determined. Having small sample sizes can impact on the accuracy of these genetic diagnostic rates, as demonstrated with the ASDA subgroup; for the Oculome panel test 24.8% (28 of 113 cases) received a molecular diagnosis, but from our clinic this reached 60% (6/10) cases solved, however the larger WGS cohort of 46 patients found only 13.0% (six cases) were solved, demonstrating the variability in outcomes. Conversely, in the congenital cataract group undergoing the Oculome panel test, eight of nine cases (88.9%) received a molecular diagnosis. We had a similar number of patients from our clinics but only solved four out of nine families (44.4%), and with WGS 44.7% (17/38) were solved.

For MAC conditions, the Oculome panel test was able to solve 8.2% of cases but through our clinics and the 100,000 Genomes Project (using WGS), our patients had at least a twofold increase with 21.4% (3/14) and 15.7% (11/70) diagnostic rates, respectively. This confirms that WGS has the capacity to increase diagnostic rates. The most prevalent gene was *PRSS56* (OMIM #613858), which causes autosomal recessive microphthalmia, isolated 6 (OMIM #613517), found in two unrelated families (16899 and 25356). All other MAC families had their own individual disease‐causing gene, which can make genotype–phenotype correlations hard to determine as many more cases need to be identified to strengthen associations and prognosis. As only 11 out of 70 cases were solved, this suggests that many deep intronic variants may be as yet undetected due to the diagnostic pipeline limitations. This low solve rate also implies that more variants and novel genes remain undiscovered, and/or possible alternate non‐Mendelian disease aetiologies, for example epigenetic or complex genetic causes. In a recent paper using the wildtype zebrafish as a model of optic fissure morphogenesis, ocular tissue from the site of the unfused, fusing and post‐fusion optic fissure was taken for comparative global transcriptomic profiling against dorsal retina at the same timepoint (Richardson et al., 2019). Overall, 322 differentially expressed genes were found to be involved in optic fissure morphogenesis, these can be further interrogated through knockdown/out studies for resultant MAC phenotypes, comparative analysis in other animal models looking for conserved genes, and in larger patient cohorts such as those recruited to the 100,000 Genomes Project (Caulfield et al., 2019) and the NIHR Bioresource (NBR‐RD, 2019).

The use of human induced pluripotent stem cells (hiPSCs) has been manipulated to generate models of human eye development, and could be used to identify more candidate genes by revealing underlying molecular mechanisms (Hung, Khan, Lo, Hewitt, & Wong, 2017; Llonch, Carido, & Ader, 2018). Optic vesicle‐like structures derived from a microphthalmia patient with a *VSX2* null variant (p.Arg200Gln) showed upregulation of WNT pathway components and misexpression of retinal pigment epithelium (RPE) markers at the expense of the neural retina (NR) lineage, which was rescued by pharmacological inhibition of WNT signaling (Capowski et al., 2016). This supports an important role for *VSX2* in WNT signaling and maintenance of the NR through WNT pathway suppression. One potential problem of in vitro modeling is the lack of surrounding embryological tissue and external stimuli that may provide cues for in vivo development, this is particularly important given the involvement of multiple ocular structures in patients. Utilization of data from both cell‐based systems and animal models will continue to provide a more complete and accurate representation.

The given primary diagnosis can influence the choice of gene panel and subsequent diagnostic rates. For example, in this study family 36 with congenital cataracts was found to harbor a heterozygous frameshift deletion (c.856del, p.[Ser286Alafs*92]) in *BCOR* (OMIM #300485), and this causes X‐linked dominant microphthalmia, syndromic 2 (OMIM #300166). The female patient presented with bilateral congenital cataracts, unilateral left microphthalmia and persistent primary teeth, and thus could fall into both MAC and cataract cohorts, but was counted under congenital cataract. Similarly, family 17 had unilateral left microphthalmia with cornea plana, anterior segment dysgenesis, left exotropia and high hypermetropia, and was identified to have a homozygous missense variant (c.809C>T, p.[Ser270Leu]) in the *KERA* gene (OMIM #603288). *KERA* is known to cause autosomal recessive corneal plana 2 (OMIM #217300) and this variant is rare in healthy population databases, although not previously reported to cause disease. The unaffected father was confirmed as a heterozygous carrier, and the unaffected mother did not undergo genetic testing. *KERA* is not found in the MAC targeted gene panel despite several case reports suggesting its association (Huang et al., 2019; Kumari et al., 2016; Lehmann et al., 2001). This variant was identified from WGS, which has more flexibility to screen novel genes or variants. The drawback of targeted gene panels is that they are predesigned to cover given regions of the genome, and if updates are required, a new panel must be designed which incurs costs for time and validation. In contrast, WGS covers the entire genome, over 3 billion nucleotides and 20,000 genes, and can identify previously noncovered variants such as CNVs, structural variations, intergenic and deep intronic variants (Lionel et al., 2018; Van Cauwenbergh et al., 2017; Vaz‐Drago, Custodio, & Carmo‐Fonseca, 2017). This is important for developmental eye disorders, such as aniridia where ~15% of all *PAX6* variants are located in intronic regions, with the majority at the intron–exon border (Lima Cunha, Arno, Corton, & Moosajee, 2019), although only 8.6% were noncoding variants from the WGS group in this study.

A notable finding for patients with query albinism and infantile nystagmus, was that a number of genes were found in both subgroups, including *OCA2* and *SLC38A8*. This highlights the diagnostic challenges of young children with nystagmus as their presenting complaint. There is significant phenotypic variability in albinism patients, which can make it hard to delineate from infantile nystagmus. Visual evoked potentials are excellent at detecting intracranial misrouting, but this can be inconclusive in young infants. Hence, for some patients their diagnosis will be revised based on their molecular result, for example one nystagmus family (25824) was found to have biallelic variants in *OCA2* (OMIM #611409) causing autosomal recessive oculocutaneous albinism type II (OMIM #203200). Hence further clinical phenotyping can be required to confirm the correct clinical diagnosis. Four unrelated families (56, 26350, 58, and 26364) were identified with biallelic *SLC38A8* (OMIM #615585) variants through WGS (as this gene is not present on targeted gene panels for nystagmus or albinism). It causes autosomal recessive foveal hypoplasia 2, with or without optic nerve misrouting and/or anterior segment dysgenesis (OMIM #609218), and thus its features can pose clinical diagnostic challenges. This reinforces the need to undertake genetic testing in order to reach a definitive diagnosis that can guide the ongoing care and management of the patient. As more genes are identified, it is likely that we will begin to see more extensive spectrums of disease rather than distinct disease entities. Classifications of disease using eponymous names must also be superseded by a gene‐based system where we can build our knowledge of genotype–phenotype relationships.

Patient access to genetic testing has been a barrier to accurate, timely diagnoses and appropriate management. In countries with insurance‐based systems, agencies are reluctant to fund genetic testing unless there is clear evidence the results will accurately determine the clinical status of the patient and directly influence management (Capasso, 2014). In the United Kingdom, the 100,000 Genomes Project was established to provide an infrastructure that allows NHS patients with rare disease to access WGS through a genomic medicine service with centralized funding (Patch & Middleton, 2019; Turnbull et al., 2018). This transition is underway, with designated laboratories undertaking ophthalmic genetic testing and oversight from Genomic Hubs permitting equity of genomics services (Royal College of Ophthalmologists, 2020). The National Genomic Test Directory for rare and inherited diseases has been formed detailing which test is available for each clinical indication, with the Genomics England Panel app listing the genes included in each panel, reviewed by experts to ensure there is evidence for its inclusion (Genomics England Panel App, n.d.). Such advances will accelerate time to diagnosis, improve diagnostic rates, permit precision management and cost saving on prolonged, multidisciplinary team assessments and investigations (Gillespie et al., 2014; Musleh et al., 2016). For patients and families with developmental eye disorders, an accurate molecular diagnosis earlier in the patient pathway potentially provides several benefits by addressing uncertainty, improving decision‐making and elucidation of any systemic manifestations, a number of which are can be potentially life threatening. Research into developmental eye disorders will also greatly benefit from larger solved patient datasets.

## 5 CONCLUSION

Understanding the etiology of developmental eye disorders remains challenging given their diverse phenotypes and genetic heterogeneity. Diagnostic rates remain variable and relatively lower than the progress made with IRDs. NGS technologies allow variants to be screened in parallel and at relatively low cost. Expanding WGS from the research setting to an accredited clinical service will allow for more accurate diagnosis and improved management of patients. Ensuring precise human phenotype ontology is used to document each clinical feature (not just the primary diagnosis) will enable clinical scientists to best apply the relevant diagnostic gene panel. Being able to gain a molecular diagnosis will further our understanding of the natural history of gene/variant‐specific cohorts, reveal potential therapeutic targets and establish outcome measures for prospective future treatments.

## CONFLICT OF INTEREST

The authors have no conflicts of interest to declare.

## Supporting information

**Table S1** Supporting Information.

## Data Availability

The datasets generated for this study were submitted to ClinVar (https://www.ncbi.nlm.nih.gov/clinvar/) through the Rare & Inherited Disease Genomic Laboratory at Great Ormond Street Hospital (London, UK).
