# Supplementary material for: Molecular diagnostic challenges for non‐retinal developmental eye disorders in the United Kingdom
Source: Am J Med Genet C Semin Med Genet. 2020 Aug 23;184(3):578–89. doi: 10.1002/ajmg.c.31837 (PMC8432170; doi:10.1002/ajmg.c.31837)
Supplement: Supplementary file 1 — Table S1 Supporting Information. [file AJMG-184-578-s001.docx]

| **Family ID** | **Patient ID** | **Age** | **Gender** | **Ethnicity** | **Diagnosis group** | **Clinical diagnosis**  **(prior to testing)** | **Phenotype terms** | **Test** | **Genetic results** | **Gene** |
| --- | --- | --- | --- | --- | --- | --- | --- | --- | --- | --- |
| 1 | 1-i | 4 months | M^[[1]](#footnote-1)^† | Not stated | MAC^[[2]](#footnote-2)^‡ | Kabuki Syndrome | Chorioretinal coloboma; Iris coloboma; Esodeviation; Ventricular septal defect; Patent ductus arteriosus; Congenital cystic adenomatoid malformation of the lung | Panel^[[3]](#footnote-3)^§ | Gene found | *KMT2D* |
| 2 | 2-i | 11 months | F^[[4]](#footnote-4)^¶ | Other | MAC | Microphthalmia | Unilateral microphthalmos; Morning glory anomaly; Coloboma; Retinal detachment | WGS^[[5]](#footnote-5)^† | No primary findings |  |
| 3 | 3-i | 11 years | F | White British | MAC | Unilateral Anophthalmia | True anophthalmia | WGS | No primary findings |  |
| 4 | 4-i | 5 years | F | Asian Indian | MAC | Microphthalmia | Unilateral microphthalmos | WGS | Gene found | *MAB21L2* |
| 5 | 5-i | 5 years | F | Not stated | MAC | Chorioretinal Coloboma | Chorioretinal coloboma; Iris coloboma; Hypoplasia of the iris; Persistent pupillary membrane | Panel | No primary findings |  |
| 6 | 6-i | 4 years | M | Other | MAC | Ocular Coloboma | Chorioretinal coloboma; Chorioretinal atrophy; Decreased circulating cortisol level; Exotropia; Hypermetropia; Astigmatism | WGS | No primary findings |  |
| 7 | 7-i | 1 month | F | White Other | MAC | Chorioretinal Coloboma | Chorioretinal coloboma; Unilateral microphthalmos; Abnormality of the optic disc | WGS | No primary findings |  |
| 8 | 8-i | 5 years | M | White Other | MAC | Microphthalmia | Unilateral microphthalmos; Intellectual disability | WGS | No primary findings |  |
| 9 | 9-i | 2 years | F | Asian Pakistani | MAC | Bilateral severe microphthalmia | Bilateral microphthalmos | WGS | Gene found | *ALDH1A3* |
| 10 | 10-i | 1 year | M | Black African | MAC | Microphthalmia | Unilateral microphthalmos; Cataract | WGS | No primary findings |  |
| 11 | 11-i | 1 month | F | White Other | MAC | Microphthalmia | Bilateral microphthalmos; Chorioretinal coloboma; Retinal detachment; Hydronephrosis; Intrauterine growth retardation | WGS | No primary findings |  |
| 12 | 12-i | 1 year | M | White British | MAC | Optic Disc Anomaly | Optic disc anomaly | WGS | No primary findings |  |
| 13 | 13-i | 11 months | F | White British | MAC | Bilateral Microphthalmia, Iris and Chorioretinal Coloboma | Bilateral microphthalmos; Iris coloboma; Chorioretinal coloboma; Nephrosclerosis; Recurrent urinary tract infections | WGS | No primary findings |  |
| 14 | 14-i | 8 months | M | Other | MAC | Microphthalmia | Unilateral microphthalmos; Toe syndactyly | NA^[[6]](#footnote-6)^‡ - family declined | NA |  |
| 15 | 15-i | 10 years | M | White British | MAC | Chorioretinal Coloboma | Chorioretinal coloboma; Abnormality of the optic disc | Panel | No primary findings |  |
| 16 | 16-i | 2 years | M | White British | ASDA^[[7]](#footnote-7)^§ | Congenital Hereditary Endothelial Dystrophy | Congenital corneal dystrophy | Panel | Gene found | *SLC4A11* |
| 17 | 17-i | 3 years | M | Asian Other | ASDA | Anterior Segment Dysgenesis | Anterior segment dysgenesis; Sclerocornea; Microcornea; Microphthalmos, High hypermetropia | WGS | Gene found | *KERA* |
| 17 | 17-ii | 5 years | M | Asian Other | ASDA | Anterior Segment Dysgenesis | Anterior segment dysgenesis; Microcornea; Microphthalmos, High hypermetropia; Exotropia; Conical tooth | WGS | Gene found | *KERA* |
| 18 | 18-i | 9 years | F | White Other | ASDA | Axenfeld-Rieger Syndrome | Posterior embryotoxon; Aplasia/Hypoplasia of the iris; Iris transillumination defect; High hypermetropia; Esodeviation | WGS | No primary findings |  |
| 19 | 19-i | 2 months | F | Asian Indian | ASDA | Anterior Segment Dysgenesis | Anterior segment dysgenesis; Unilateral microphthalmos; Iris coloboma; Retinal coloboma | NA - unable to obtain sample | NA |  |
| 20 | 20-i | 14 years | F | Not stated | ASDA | Aniridia | Aniridia; Intellectual disability | Single gene | Gene found | *PAX6* |
| 21 | 21-i | 9 years | F | Asian Pakistani | ASDA | Cornea Plana | Cornea Plana; High hypermetropia; Central opacification of the cornea; Amblyopia | Panel | Gene found | *KERA* |
| 21 | 21-ii | 7 years | M | Asian Pakistani | ASDA | Cornea Plana | Cornea Plana; High hypermetropia; Central opacification of the cornea; Amblyopia | Panel | Gene found | *KERA* |
| 22 | 22-i | 6 years | F | Black Other | ASDA | Reis-Buckler Corneal Dystrophy | Granular corneal dystrophy | Panel | Gene found | *TGFB1* |
| 23 | 23-i | 1 year | M | Other | ASDA | Bilateral ectrodactyly-ectodermal dysplasia-clefting (EEC) syndrome with limbal stem cell deficiency | Abnormal corneal epithelium morphology; Band keratopathy; Limbal stem cell deficiency; Keratitis; Ectodermal dysplasia; Alopecia of scalp | WGS | No primary findings |  |
| 24 | 24-i | 1 year | M | White Other | ASDA | Congenital Glaucoma | Primary congenital glaucoma | WGS | No primary findings |  |
| 25 | 25-i | 10 years | M | White British | ASDA | Juvenille Open Angle Glaucoma | Open angle glaucoma | Panel | Gene found | *MYOC* |
| 25 | 25-ii | 13 years | M | White British | ASDA | Juvenille Open Angle Glaucoma | Open angle glaucoma | Panel | Gene found | *MYOC* |
| 26 | 26-i | 1 year | F | Not stated | ASDA | Congenital Glaucoma | Primary congenital glaucoma; Opacification of the corneal stroma; Intrauterine growth retardation; Microcephaly; Pulmonic stenosis; Right ventricular hypertrophy; Heart murmur; Hearing impairment | Panel | No primary findings |  |
| 27 | 27-i | 4 years | F | Asian Other | Cataract | Blue Dot Cataract | Cerulean cataract; Myopia; Astigmatism | WGS | Gene found | *CRYBB2* |
| 27 | 27-ii | 4 months | F | Asian Other | Cataract | Blue Dot Cataract | Cerulean cataract; Hypermetropia; Astigmatism | WGS | Gene found | *CRYBB2* |
| 27 | 27-iii | 10 years | M | Asian Other | Cataract | Blue Dot Cataract | Cerulean cataract; Hypermetropia; Astigmatism | WGS | Gene found | *CRYBB2* |
| 28 | 28-i | 14 years | M | White British | Cataract | Congenital Cataract | Developmental cataract; Nystagmus; Hypermetropia | WGS | Gene found | *EPHA2* |
| 28 | 28-ii | 4 years | M | White British | Cataract | Congenital Cataract | Developmental cataract; Nystagmus; Hypermetropia | WGS | Gene found | *EPHA2* |
| 28 | 28-iii | 8 years | M | White British | Cataract | Congenital Cataract | Developmental cataract; Nystagmus; Hypermetropia | WGS | Gene found | *EPHA2* |
| 29 | 29-i | 10 months | M | Asian Bangladeshi | Cataract | Congenital Cataract | Developmental cataract; Pendular nystagmus | WGS | No primary findings |  |
| 30 | 30-i | 1 year | F | Not stated | Cataract | Congenital Cataract | Developmental cataract; Esotropia; Amblyopia | NA - family declined | NA |  |
| 31 | 31-i | 2 months | F | Mixed White/Black African | Cataract | Polar cataract | Anterior polar cataract; Congenital hypertrophy of retinal pigment epithelium | WGS | No primary findings |  |
| 32 | 32-i | 7 months | M | Other | Cataract | Congenital Cataract | Developmental cataract; Congenital aphakia | WGS | Gene found | *HSF4* |
| 33 | 33-i | 14 years | M | White British | Cataract | Blue Dot Cataract | Cerulean cataract; Nuclear cataract; Myopia; Attention deficit hyperactivity disorder | WGS | No primary findings |  |
| 34 | 34-i | 3 years | F | Black Other | Cataract | Congenital Cataract | Developmental cataract; Esotropia; Biventricular hypertrophy; Hyperinsulinemic hypoglycemia | Panel | Results pending |  |
| 35 | 35-i | 11 years | M | Asian Bangladeshi | Cataract | Congenital Cataract | Developmental cataract; Pseudophakia; Intellectual disability; Headache; Overweight; Neonatal jaundice | WGS | Results pending |  |
| 36 | 36-i | 13 years | F | White British | Cataract | Congenital Cataract | Developmental cataract; Unilateral microphthalmos; Congenital aphakia; Iris cyst; Subconjunctival hemorrhage; Band keratopathy | WGS | Gene found | *BCOR* |
| 37 | 37-i | 2 years | F | Other | Nystagmus | Infantile Nystagmus | Nystagmus; Exotropia; Bilateral ptosis; Global developmental delay | WGS | No primary findings |  |
| 38 | 38-i | 10 years | M | White British | Nystagmus | Infantile Nystagmus | Nystagmus; Ventricular septal defect | WGS | No primary findings |  |
| 39 | 39-i | 7 years | F | White Other | Nystagmus | Infantile Nystagmus | Nystagmus; Hypermetropia | WGS | No primary findings |  |
| 40 | 40-i | 10 years | M | Mixed White/Black African | Nystagmus | Infantile Nystagmus | Nystagmus; Mild hypermetropia; Short attention span; Intellectual disability; Abnormality of coordination | WGS | Gene found | *CACNA1A* |
| 41 | 41-i | 9 months | M | Other | Nystagmus | Congenital Nystagmus | Congenital horizontal nystagmus | WGS | No primary findings |  |
| 42 | 42-i | 6 years | M | White Other | Nystagmus | Congenital Nystagmus | Congenital nystagmus; Hyperopic astigmatism | WGS | No primary findings |  |
| 43 | 43-i | 2 years | M | White Other | Nystagmus | Infantile Nystagmus | Congenital horizontal nystagmus | WGS | No primary findings |  |
| 44 | 44-i | 4 years | F | White Other | Nystagmus | Infantile Nystagmus | Nystagmus | WGS | No primary findings |  |
| 45 | 45-i | 2 years | F | Other | Nystagmus | Infantile Nystagmus | Upbeat nystagmus; Astigmatism | WGS | No primary findings |  |
| 46 | 46-i | 11 years | M | Other | Nystagmus | X-linked Congenital Nystagmus | Pendular nystagmus; Hypermetropia; Astigmatism | WGS | Gene found | *FRMD7* |
| 47 | 47-i | 1 year | M | White British | Nystagmus | Congenital Nystagmus | Congenital nystagmus | WGS | No primary findings |  |
| 48 | 48-i | 5 years | M | Not stated | Nystagmus | Infantile Nystagmus | Nystagmus; Premature birth; Esotropia | Panel | No primary findings |  |
| 49 | 49-i | 3 years | M | White British | Nystagmus | Congenital Nystagmus | Congenital nystagmus; Premature birth; Multiple bilateral pneumothoraxes | WGS | No primary findings |  |
| 49 | 49-ii | 3 years | M | White British | Nystagmus | Congenital Nystagmus | Congenital nystagmus; Premature birth | WGS | No primary findings |  |
| 50 | 50-i | 5 years | M | Black African | Albinism | Oculocutaneous Albinism | Ocular albinism; Horizontal nystagmus; Absent foveal reflex; Hypoplasia of the fovea | WGS | No primary findings |  |
| 51 | 51-i | 7 months | F | White British | Albinism | Oculocutaneous Albinism | Ocular albinism; Nystagmus; Alternating esotropia; Hypermetropia; Astigmatism | WGS | Gene found | *OCA2* |
| 52 | 52-i | 10 years | M | Black Caribbean | Albinism | Ocular Albinism | Ocular albinism; Congenital nystagmus; Exotropia; Astigmatism | WGS | Gene found | *GPR143* |
| 53 | 53-i | 2 years | F | Other | Albinism | Hermansky-Pudlak Syndrome | Ocular albinism; Nystagmus; Partially accommodative esotropia; Hypermetropia; Astigmatism | WGS | Gene found | *HPS6* |
| 53 | 53-ii | 5 years | F | Other | Albinism | Oculocutaneous Albinism | Ocular albinism; Hypoplasia of the fovea; Exodeviation; Hypermetropia; Astigmatism | WGS | Gene found | *HPS6* |
| 54 | 54-i | 15 years | M | White Other | Albinism | Ocular Albinism | Ocular albinism; Horizontal jerk nystagmus; Hyperopic astigmatism | WGS | No primary findings |  |
| 55 | 55-i | 6 years | F | Asian Bangladeshi | Albinism | Ocular Albinism | Ocular albinism; Congenital nystagmus; Hypermetropia; Astigmatism | WGS | No primary findings |  |
| 56 | 56-i | 2 years | F | White British | Albinism | Ocular Albinism | Ocular albinism; Nystagmus; Hypoplasia of the fovea | WGS | Gene found | *SLC38A8* |
| 57 | 57-i | 11 years | F | Asian Other | Albinism | Oculocutaneous Albinism | Ocular albinism; Consecutive exotropia; Nystagmus; Astigmatism | WGS | Results pending |  |
| 58 | 58-i | 2 years | M | Asian Indian | Albinism | Ocular Albinism | Ocular albinism; Nystagmus; Hypoplasia of the fovea; Optic nerve misrouting | WGS | Gene found | *SLC38A8* |
| 59 | 59-i | 3 years | M | White Other | Strabismus | Blepharophimosis, ptosis, and epicanthus inversus syndrome | Blepharophimosis; Bilateral ptosis | Panel | No primary findings |  |
| 60 | 60-i | 7 years | M | Not stated | Strabismus | Duane's Retraction Syndrome | Duane anomaly; Hypermetropia; Esotropia; Global developmental delay; Macroglossia; Speech apraxia | Panel | No primary findings |  |
| 61 | 61-i | 2 years | M | Asian Pakistani | Strabismus | Congenital Fibrosis of Extra Ocular Muscles | Congenital fibrosis of extraocular muscles; Congenital bilateral ptosis | WGS | No primary findings |  |
| 61 | 61-ii | 3 years | M | Asian Pakistani | Strabismus | Congenital Fibrosis of Extra Ocular Muscles | Congenital fibrosis of extraocular muscles; Congenital bilateral ptosis; Amblyopia; Strabismus | WGS | No primary findings |  |
| 62 | 62-i | 3 years | M | Not stated | Strabismus | Congenital Fibrosis of Extra Ocular Muscles | Congenital fibrosis of extraocular muscles; Congenital bilateral ptosis; Abnormality of eye movement; Esodeviation; Astigmatism; Amblyopia | Panel | No primary findings |  |

**Supplemental Table 1.** All demographic, clinical and genetic details from 72 consecutive patients with non-retinal developmental eye disorders presenting to the paediatric ocular genetics service between 1^st^ October 2017 to 30^th^ September 2018.

1. †M – Male [↑](#footnote-ref-1)
2. ‡MAC – Microphthalmia, anophthalmia and coloboma [↑](#footnote-ref-2)
3. §Panel – Targeted gene panel [↑](#footnote-ref-3)
4. ¶F – Female [↑](#footnote-ref-4)
5. †WGS – Whole genome sequencing [↑](#footnote-ref-5)
6. ‡NA – Not available [↑](#footnote-ref-6)
7. §ASDA – Anterior segment dysgenesis anomalies [↑](#footnote-ref-7)
